# Combined Single‐Cell and Spatial Transcriptomics Reveal the Metabolic Evolvement of Breast Cancer during Early Dissemination

**DOI:** 10.1002/advs.202205395

**Published:** 2023-01-03

**Authors:** Yi‐Ming Liu, Jing‐Yu Ge, Yu‐Fei Chen, Tong Liu, Lie Chen, Cui‐Cui Liu, Ding Ma, Yi‐Yu Chen, Yu‐Wen Cai, Ying‐Ying Xu, Zhi‐Ming Shao, Ke‐Da Yu

**Affiliations:** ^1^ Department of Breast Surgery Shanghai Cancer Center and Cancer Institute Fudan University Shanghai 200032 P. R. China; ^2^ Shanghai Medical College Fudan University Shanghai 200032 P. R. China; ^3^ Department of Breast Surgery Harbin Medical University Cancer Hospital Harbin Heilongjiang 150081 P. R. China; ^4^ Department of Breast Surgery The First Affiliated Hospital of China Medical University Shenyang Liaoning 110000 P. R. China; ^5^ Key Laboratory of Breast Cancer in Shanghai Shanghai 200032 P. R. China

**Keywords:** breast cancer, early dissemination, metabolism, single‐cell RNA sequencing, spatial transcriptomics

## Abstract

Breast cancer is now the most frequently diagnosed malignancy, and metastasis remains the leading cause of death in breast cancer. However, little is known about the dynamic changes during the evolvement of dissemination. In this study, 65 968 cells from four patients with breast cancer and paired metastatic axillary lymph nodes are profiled using single‐cell RNA sequencing (scRNA‐seq) and spatial transcriptomics. A disseminated cancer cell cluster with high levels of oxidative phosphorylation (OXPHOS), including the upregulation of cytochrome C oxidase subunit 6C and dehydrogenase/reductase 2, is identified. The transition between glycolysis and OXPHOS when dissemination initiates is noticed. Furthermore, this distinct cell cluster is distributed along the tumor's leading edge. The findings here are verified in three different cohorts of breast cancer patients and an external scRNA‐seq dataset, which includes eight patients with breast cancer and paired metastatic axillary lymph nodes. This work describes the dynamic metabolic evolvement of early disseminated breast cancer and reveals a switch between glycolysis and OXPHOS in breast cancer cells as the early event during lymph node metastasis.

## Graphical abstract

By single‐cell RNA sequencing and spatial transcriptomics, the early early‐disseminated breast cancer cells are found to travel from the border of primary tumor to axillary lymph nodes. During this metastasis, a switch between glycolysis and oxidative phosphorylation occurs in early disseminated breast cancer cells, indicating an interesting dynamic metabolic evolvement.

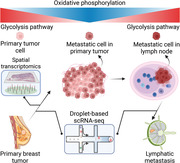

## 1 Introduction

Breast cancer has become the most common malignancy worldwide, accounting for nearly 30% of female cancers.^[^ ^1^ ^]^ Owing to the rapid development of therapeutic approaches, most patients with non‐metastatic breast cancer are curable. However, advanced breast cancers with distant metastasis are still considered to be incurable. Axillary lymph nodes are the most common site for metastasis. The status of axillary lymph nodes is a crucial clinical factor during the determination of therapeutic strategies and is also of critical significance in risk estimation and prognosis evaluation.^[^ ^2^ ^]^

Single‐cell RNA sequencing (scRNA‐seq) is a powerful tool that can provide expression profiling of human cancer at the resolution of individual cells, which allows the identification and characterization of specific subclusters that bear unique biological effects.^[^ ^3^ ^]^ Such studies have been widely performed in breast cancer research to investigate the tumor microenvironment (TME) and the evolvement of tumor cells.^[^ ^4^, ^5^, ^6^, ^7^, ^8^, ^9^, ^10^, ^11^ ^]^ It has been gaining significant interest that the spatial arrangement and cellular interactions can affect the state of cells to a massive extent; however, information on the location was not preserved when using scRNA‐seq to measure the state of cells.^[^ ^12^ ^]^ Spatial transcriptomics (ST) is a novel biotechnology that allows visualization and quantitative analysis of the transcriptome with spatial resolution in tissue sections, compensating for the lack of spatial information in scRNA‐seq.^[^ ^13^ ^]^ Combining ST and scRNA‐seq helps to overcome the limitations of both scRNA‐seq—which lacks spatial information—and ST—which is not at individual cell resolution.^[^ ^14^ ^]^ Multimodal intersection analysis (MIA) is one of the useful approaches which provides meaningful biological insight into research by combining ST and scRNA‐seq.^[^ ^15^ ^]^ Regional lymph nodes are often the earliest sites of metastasis when breast cancer cells leave the primary tumor to the whole body.^[^ ^16^, ^17^ ^]^ Therefore, we used patients with breast cancer and paired metastatic axillary lymph nodes to mimic the early dissemination events. By integrating these two complementary technologies, we intended to provide an insight into the process during early dissemination.

In this study, we applied scRNA‐seq and discovered a cluster of breast cancer cells that disseminated. This distinct cell cluster exhibited upregulated oxidative phosphorylation (OXPHOS) pathway. We found that this cluster experienced a reverting transition between OXPHOS and glycolysis using pseudo‐time analysis. CellChat analysis was also conducted to map its cellular interaction with immune cells. Furthermore, we also performed ST analysis to identify that this disseminated cell cluster is located along the tumor's leading edge. We tested these findings via cytological experiments and immunohistochemical staining and verified the results in both bulk and single‐cell RNA‐seq cohorts.

## 2 Results

### 2.1 scRNA‐Seq Identified Early‐Disseminated Cancer Cell Clusters in Paired Primary Breast Tumors and Metastatic Lymph Nodes

**Figure 1:**
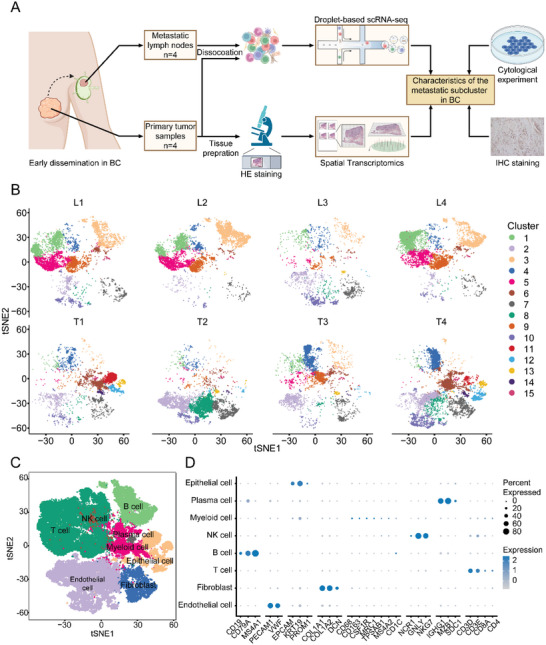
Expression profiling of 65 968 single cells in paired primary breast tumors and metastatic lymph nodes. A) Graphic overview of this study design. Tumor and paired lymph node tissue from four breast cancer patients were processed into single‐cell suspension and unsorted cells were used for scRNA‐seq with 10x Genomics. Tumor slides were processed to obtain by 10x Genomics Visium. The following integrated analysis of cytological experiments and IHC staining is described in squares. B) t‐SNE plots of 65 968 cells from tumor and paired lymph node tissue of four breast cancer patients, showing 15 clusters in each plot. Each cluster is shown in a different color. C) t‐SNE plot of 65 968 cells profiled in the present study colored by major cell types. D) Bubble plots of the marker genes expressed in the major cell types. Dot color reflects expression level and dot size represents the percent of cells expressing marker genes in different cell types. IHC, immunohistochemistry. HE, hematoxylin and eosin. scRNA‐seq, single‐cell RNA sequencing. t‐SNE, t‐distributed stochastic neighbor embedding.

To comprehensively identify the cellular composition and architecture in primary tumors and metastatic lymph nodes, we performed scRNA‐seq (10x Genomics) on four pairs of primary invasive breast tumors and metastatic axillary lymph nodes collected from four patients in Fudan University Shanghai Cancer Center (FUSCC) cohort during surgical resection (**Figure** **1**A and Data S1, Supporting Information). Two pathologists confirmed all lymph nodes to ensure metastasis. After filtering the scRNA‐seq data to exclude damaged or dead cells and putative cell doublets, we constructed an atlas consisting of 65 968 single cells with a median of 1437 expressed genes that passed the stringent quality filtering (see Experimental Section).

After integrating the transcriptional data from all acquired cells, we first applied low‐resolution t‐distributed stochastic neighbor embedding (t‐SNE) clustering and generated 2D graphs with 15 clusters in eight samples (Figure 1B). To identify the main cell types of this atlas, we annotated each cluster with their marker gene expression (Figure 1C and Figure S1A,B, Supporting Information). All cells were divided into eight cell types, including epithelial cells (ECAM1, KRT19, PROM1), plasma cells (IGHG1, MZB1, SDC1), myeloid cells (CD68, CD163, CSF1R, MRC1, TPSAB1, MS4A2, CD1C), natural killer (NK) cells (NCR1, GLNY, NKG7), B cells (CD79A, CD79B, MS4A1), T cells (CD3D, CD3E, CD8A, CD4), fibroblasts (COL1A1, COL1A2, DCN), and endothelial cells (PCAM1, VWF) (Figure 1D and Figure S1C and Data S2, Supporting Information).

**Figure 2:**
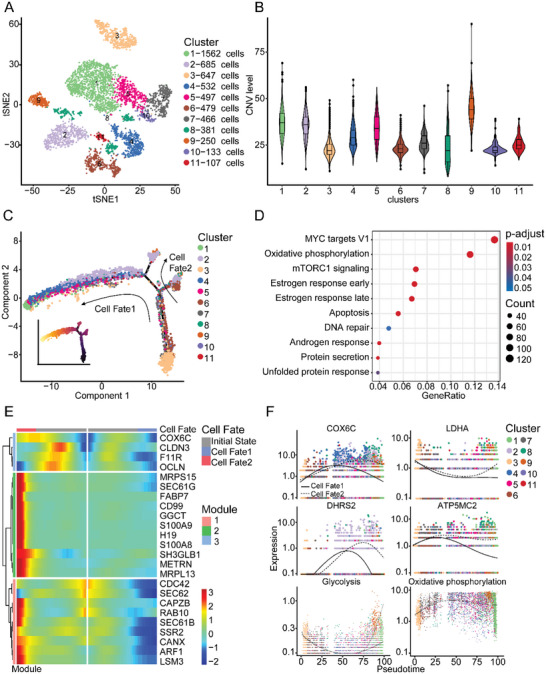
Metastatic epithelial cell characteristics identified by scRNA‐seq. A) t‐SNE plot of 5739 epithelial cells showing 11 clusters. Each cluster is shown in a different color. B) Violin plots of the CNV levels in 11 epithelial cell clusters. C) Potential trajectory of all epithelial cells identified two distinct cell fates colored by cluster. The arrow shows the potential evolutionary direction in the trajectory. D) Bubble plot of hallmarks for DEGs between EDC clusters and other epithelial cell clusters. The intensity represents the adjusted *p*‐value of each hallmark. Dot size shows gene count for each hallmark. Wilcoxon signed‐rank test was used to assess the difference. E) Heatmap showing selected DEGs (rows) between EDC clusters and other epithelial cell clusters along the pseudo‐time (columns), which was clustered into three profiles. Color key differentially coding from blue to red indicated the relative expression levels from low to high. F) Dot plots of dynamic expression of key genes in glycolysis and OXPHOS and the two pathways themselves based on KEGG database along two cell fates. CNV, copy number variation. DEG, differentially expressed genes. EDC, early‐disseminated cancer cell. KEGG, Kyoto Encyclopedia of Genes and Genomes. OXPHOS, oxidative phosphorylation. Pre‐branch, premalignant‐status branch.

Given that breast cancer cells originate from epithelial cells, we then performed high‐resolution t‐SNE analysis and re‐cluster the epithelial cells into 11 clusters (**Figure** **2**A and Figure S2A,B, Supporting Information). Copy number variation (CNV) analysis has been widely used in scRNA‐seq to investigate disease evolvement and development.^[^ ^18^, ^19^ ^]^ To distinguish malignant from non‐malignant clusters, we evaluated the CNV level of all epithelial cells and each epithelial cell cluster (Figure 2B and Figure S2C,D, Supporting Information). As expected, the CNV levels of epithelial cells in lymph nodes were significantly higher than those in primary tumors (Figure S2E, Supporting Information). Among all epithelial cell clusters, C3 exhibited remarkably lower CNV levels than other clusters (Figure 2B), suggesting C3 is a group of normal mammary ductal epithelial cells, and other clusters are malignant epithelial cells. Based on the transformation of CNV along the metastasis of breast cancer cells, we constructed four evolutionary trees of epithelial cells for each patient (Figure S3A, Supporting Information). All evolutionary trees showed a similar evolutionary trajectory from the primary tumor to lymph node, indicating the common features of tumor evolution during the early dissemination of breast cancer. To dissect the evolutionary dynamics of breast epithelial lineages, we also constructed a pseudo‐time cell trajectory analysis of the 11 epithelial cell clusters and generated a four‐branch trajectory depicting the development from non‐malignant, malignant, to metastatic cells (Figure 2C). Due to its low CNV level, C3 was located at the bottom‐right corner of the trajectory curve, suggesting the clear starting point of this evolving trajectory curve. After confirmation of this starting point, developmental routes were determined to begin with the initial state and then bifurcating into either Cell fate 1 or Cell fate 2 branches with metastasis‐rich endpoints (Figure 2C and Figure S3B, Supporting Information). Hence, C2, 4, and 9 were identified as early‐disseminated cancer cell (EDC) clusters since they were at the end of the trajectory curve with relatively high CNV levels (Figure 2B,C). More importantly, these three clusters appeared in both primary tumors and lymph nodes (Figure S2B, Supporting Information). Although C8 had a CNV level close to the normal epithelial cell C3 and was part of the initial state, it was still identified as an EDC cluster due to its existence in both primary tumors and lymph nodes (Figure S2B, Supporting Information). In a word, we identified that C3 represented the normal mammary ductal epithelial cells, while C2, 4, 8, and 9 were the EDC clusters of breast cancer.

### 2.2 Metabolic Switch between OXPHOS and Glycolysis Acts as a Potential Key Regulator in EDC Clusters

To investigate the changes in the gene expression of EDC clusters (C2, 4, 8, and 9), we performed enrichment analysis based on hallmark gene sets of the Molecular Signatures Database (MsigDB)^[^ ^20^ ^]^ to identify the altering pathways in cancer cells during the disseminating process (Data S3, Supporting Information). The top enriched pathways included pathways that are associated with cell proliferation (mTORC1 signaling, MYC targets V1) and therapy response of breast cancer (estrogen response early, estrogen response late) (Figure 2D), which are compatible with the patients’ clinicopathological features and more malignant traits of EDC clusters. Interestingly, the EDC clusters displayed significant enrichment of the OXPHOS pathway, suggesting the novel metabolic traits of disseminated cells (Figure 2D and Data S3, Supporting Information). However, when analyzing each EDC cluster separately, the OXPHOS pathway was not significantly enriched in C4. Even so, the adjusted *p*‐value was close to 0.05 (Data S3, Supporting Information).

The heatmap showed how the expression of genes changed from the initial state to Cell fate 1 or 2 and identified three different transformation patterns (Figure 2E and Figure S3C–E, Supporting Information). The importance of the OXPHOS pathway in breast cancer metastasis was emphasized in the previous work of Davis et al., where switching from glycolysis to OXPHOS promotes metastasis specifically in the tumor seeding step.^[^ ^21^ ^]^ Consistent with their discoveries, this metabolic switch in our study through pseudo‐time analysis was also observed in our study. Marker genes in OXPHOS (cytochrome C oxidase subunit 6C [COX6C], dehydrogenase/reductase 2 [DHRS2], ATP5MC2, and NDUFB4) exhibited an up‐then‐down expression pattern, while marker genes in glycolysis pathways (GAPDH, LDHA, PKM, and PGK1) exhibited a down‐then‐up expression pattern during early dissemination of malignant epithelial cells. Meanwhile, the expression of genes linked to cell adhesion and migration (CLDN3, F11R) also exhibited a down‐then‐up pattern, which indicated the dynamic change of cellular behavior during the process of dissemination (Figure 2F and Figure S3F,G, Supporting Information). Herein we raised a hypothesis that this metabolic switch of breast cancer cells resembles changes in epithelial–mesenchymal transition (EMT) during the metastatic process, that is, after detachment from the primary tumor, metabolic profiles of EDCs switched from glycolysis to OXPHOS. Once the metastatic cells seed, the metabolic preference then reverts to promoting cell proliferation. Taken together, our findings revealed the transition between OXPHOS and glycolysis in the evolvement during early dissemination of breast cancer, suggesting OXPHOS might play a vital role in the metastatic process.

### 2.3 Intercellular Interaction between EDCs and Immune Cells in the TME

**Figure 3:**
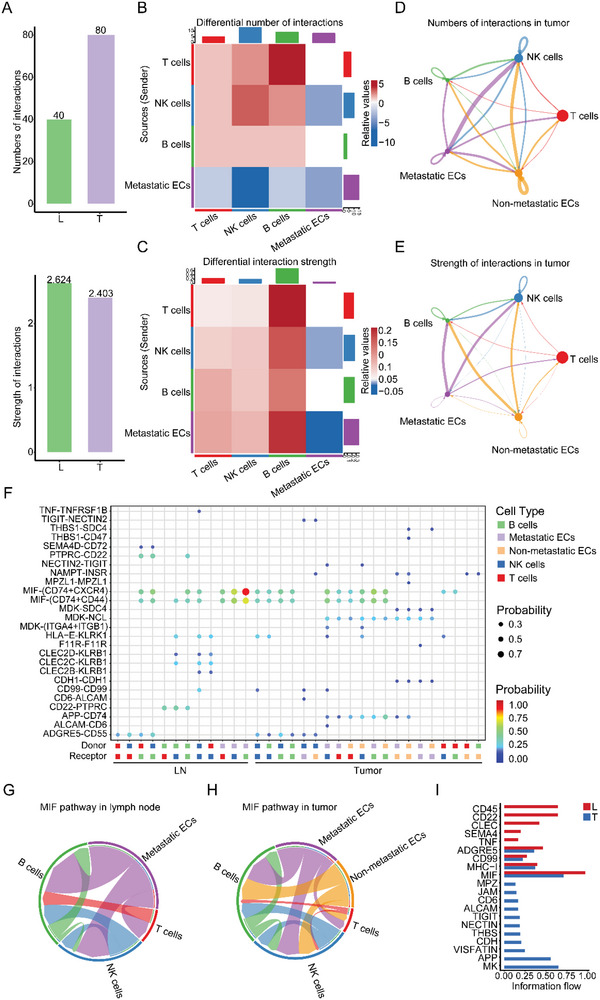
Intercellular ligand–receptor prediction among EDCs and immune cells revealed by CellChat. A) Bar plot showing the number and strength of intercellular interactions in both lymph node and tumor. B,C) Heatmaps of differential number (B) and strength (C) of intercellular interactions between lymph node and tumor. D,E) An overview of cell–cell interactions. Arrow and edge color indicate direction. Circle size is proportional to the number of cells in each cell group. Edge thickness indicates the number (D) and the strength (E) of interaction between populations. The loops indicate cell types. F) Bubble plots of the significant differentially expressed ligand–receptor pairs in the lymph node versus tumor. Dot color reflects communication probabilities, and dot size represents computed *p*‐values. Each cell group is shown in different color. Empty space means the communication probability is zero. *p*‐values are computed from a two‐sided permutation test. G,H) Chord diagrams of the inferred MIF signaling networks in lymph node (G) and tumor (H). Arc length represents the number of cells in each cell group and edge width represents the communication probability. I) Bar plots of ranked significant signaling pathways based on differences in the overall information flow within the inferred networks between lymph node and tumor. The top signaling pathways colored red are enriched in lymph node, and these colored blue pathways were enriched in the tumor. EC, epithelial cell. MIF, macrophage migration inhibitory factor.

To determine the potential interactions between EDCs and immune cells in the TME, we performed CellChat analysis to acquire cell–cell signaling links.^[^ ^22^ ^]^ Although numbers of interactions between major cell types were higher in the primary tumor, the strength of interactions between the two groups was very close (**Figure** **3**A). EDC clusters showed more interaction with immune cells in the primary tumor, while showing stronger interaction with immune cells in lymph nodes (Figure 3B–E).

We investigated the specific ligand–receptor interactions among different cell clusters. The ligand–receptor pairs among immune cells (CD22‐PTPRC, PTPRC‐CD22, and ADGRE5‐CD55) were significantly upregulated in lymph nodes versus primary tumors, suggesting that these pathways are essential for the immune response against tumors (Figure 3F and Data S4, Supporting Information). Macrophage migration inhibitory factor (MIF) pathway was active between EDCs and immune cells in both lymph nodes and primary tumors, indicating it is essential in both sites and likely to contribute to breast cancer dissemination (Figure 3F–H). We also calculated the information flows across lymph nodes and primary tumors, which is defined as the overall communication probability among all the pairs of cell groups in the communication network. Intriguingly, 4 out of 20 pathways are highly active both in lymph nodes and primary tumors, albeit at different levels (Figure 3I). These are likely the major signaling pathways between epithelial cells and immune cells independent of different TMEs. We also found 5 pathways that were specifically active in lymph nodes, while the other 11 pathways involved in apoptosis, cell migration, and proliferation were explicitly active in primary tumors, such as JAM, APP, CDH, NECTIN, and MK (Figure 3I). Altogether, CellChat analysis demonstrated that the intercellular interactions in TME were shaped in early disseminated breast cancer.

### 2.4 Spatial Transcriptome Combined with scRNA‐Seq Revealed Spatial Features of EDC Clusters

**Figure 4:**
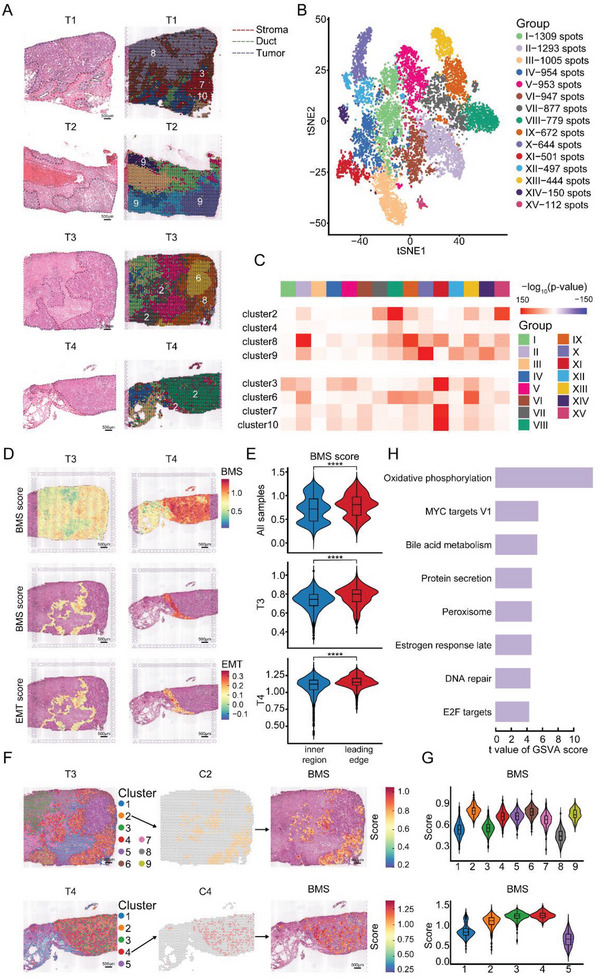
Leading edge heterogeneity revealed by ST. A) H&E staining of tissue sections (left) and mapped with unbiased clustering of ST spots in 4 tumor samples (right). Each region is surrounded by dotted lines in different color. Scale bar = 500 µm. B) t‐SNE plot of 11 137 ST spots from four primary breast cancer ST data. Each cluster is shown in different color. C) MIA map of overlap between all scRNA‐seq‐identified epithelial cell clusters and ST‐identified spot clusters. Each element in the matrix is computed for all pairs of epithelial cell clusters and spot clusters using the same 728 background genes. Red indicates enrichment (significantly high overlap); blue indicates depletion (significantly low overlap). D) All ST spots of BMS score and the tumor leading edge ST spots of BMS and EMT score in T3 and T4 tissue sections. The intensity represents score of each ST spot. Scale bar = 500 µm. E) Violin spots of BMS score of the tumor inner region and the tumor leading edge in T3, T4, and four samples together. F) Unbiased clustering of ST spots mapped to T3 and T4 tissue sections, respectively (left, middle). BMS score feature plots from T3 and T4 with data generated using the Visium ST platform. The intensity represents max expression of each gene. Scale bar = 500 µm. (right). G) Violin plots of BMS score by ST spots subpopulation in ST data. H) Bar plots of GSVA analysis comparing between leading edge‐associated ST plots and non‐leading edge‐associated ST plots in four primary breast tumor samples. All *p*‐values were determined using an unpaired two‐sided Wilcoxon rank‐sum test. ns, *p* ≥ 0.05; *, *p* < 0.05; **, *p* < 0.01; ***, *p* < 0.001; ****, *p* < 0.0001. BMS, breast metastatic signature. COX6C, cytochrome C Oxidase Subunit 6C. DHRS2, dehydrogenase/Reductase 2. EMT, epithelial–mesenchymal transition. GSVA, Gene set variation analysis. H&E, Hematoxylin and eosin. MIA, multimodal intersection analysis. ST spatial transcriptomics

To further assess the spatial organization of epithelial cells, we generated breast cancer tissue cryosections originating from the fresh tumor samples of four patients (see Experimental Section). After hematoxylin and eosin (H&E) staining and brightfield imaging, we annotated the slide into three main regions, including the tumor region, ductal epithelium, and the stromal region (**Figure** **4**A, left). We then conducted ST analysis on collected samples. Transcriptomes from 11 137 spots across four sections were obtained at a median depth of 8373 unique molecular identifiers (UMIs)/spot and 3167 genes/spot. To depict the spatial features of main cell types in the primary tumors, we mapped the scRNA data to the ST slides by SPOTlight.^[^ ^23^ ^]^ As expected, the stroma region was enriched with endothelial cells, while the tumor region was enriched with epithelial cells (Figure 4A, left, and Figure S4A and Data S5, Supporting Information). Interestingly, T cells were obviously gathered along the boundary of the stroma region and tumor region (Figure S4B, Supporting Information), suggesting the unique spatial features of this leading‐edge zone. In a word, we delineated the landscape of TME in breast primary tumors.

To explore the spatial features of EDC clusters in detail, ST data was processed using t‐SNE analysis, and all spots were divided into 15 clusters (Figure 4B). By projecting all ST clusters onto cryosections, we observed a noticeable distribution pattern, which was consistent with the histological annotations under H&E staining (Figure 4A, right). For each sample, tumor regions were made up of different clusters, including CII from T1, CX, and CXIV from T2, CIX, and CXIII from T3, CVIII, and CXV from T4. These results implied the inter‐tumor heterogeneity among four patients. To locate the epithelial cells in the samples precisely, MIA was applied to generate the corresponding relationship between the epithelial clusters and ST clusters.^[^ ^15^ ^]^ We spotted that the EDC clusters (C2, 4, 8, and 9) were enriched in the tumor region (Figures 4A, right, and 4C). Interestingly, we noticed that EDC clusters C2 and C8 were mainly located at the leading edge of the tumor regions in T3 and T4, while the other malignant cell (C6) was surrounded by C8 in T3. Together with the results from pseudo‐time analysis, we speculated that the evolvement from the malignant stage (C6) to the dissemination stage (C8) is in parallel with the route from the inside of the tumor to the leading edge and finally to metastatic sites.

To verify this hypothesis, we manually selected the spots on the tumor leading edge of each sample. Then we developed a breast metastatic signature (BMS) score based on the differential gene expression analysis between EDC clusters and other epithelial clusters (see Experimental Section). Among the top 65 genes, 26 were upregulated in the metastatic cell group (Data S6, Supporting Information). As expected, these genes are related to cell migration and cell population proliferation (e.g., HSPB1, CRIP1, and XBP1), indicating the more malignant biological feature of the EDCs cluster. Notably, the expression of multiple genes in OXPHOS pathways was also significantly altered in EDC clusters, such as COX6C, DHRS2, and NDUFC2. We also calculated the OXPHOS score and EMT score of the tumor leading edge of each sample,^[^ ^24^ ^]^ which is a known pathway associated with tumor dissemination. Although three scores varied in four samples, the tumor leading edge showed significantly higher BMS score, OXPHOS score, and EMT score than the tumor inner region in each sample (Figure 4D,E and Figure S3C–H, Supporting Information). These results suggested that the distribution of EDC clusters follows the tumor leading edge, which is consistent with the result produced by MIA and the metabolic traits of EDC clusters identified by scRNA‐seq.

To further investigate EDCs at the tumor leading edge, we performed graph‐based t‐SNE analysis on ST data of T3 and T4, re‐clustering ST spots from each sample into nine clusters and five clusters, respectively (Figure 4F, left). The two clusters (c2 in T3, c4 in T4) with the highest levels of BMS score were distributed along the tumor leading edge in both samples (Figures 4F, middle, 4F, right, and 4G). According to the results from scRNA‐seq, COX6C and DHRS2 are among the top 25 differential genes between EDC clusters and other epithelial clusters (Data S6, Supporting Information), and both participate in the OXPHOS pathway. As expected, the two clusters with the highest levels of COX6C and DHRS2 were distributed along the tumor leading edge in both samples, which is consistent with the result produced by MIA (Figure S3I, Supporting Information)

Gene set variation analysis (GSVA) was also performed to investigate differential pathways between spots along the tumor leading edge and other spots in the tumor region. The results of GSVA showed that the OXPHOS pathway was significantly upregulated in spots along the tumor leading edge. Furthermore, other enriched pathways such as DNA repair, MYC targets V1, and mTORC1 signaling were also significantly upregulated in EDC clusters (Figure 4H and Data S7, Supporting Information).

Altogether, we identified EDC clusters tended to distribute along the tumor leading edge and exhibited the metabolic signature of upregulated OXPHOS. OXPHOS might play an essential role in the metabolic transition of EDC clusters.

### 2.5 Knocking Down COX6C and DHRS2 Inhibited Breast Cancer Cells Proliferation, Migration, and EMT

**Figure 5:**
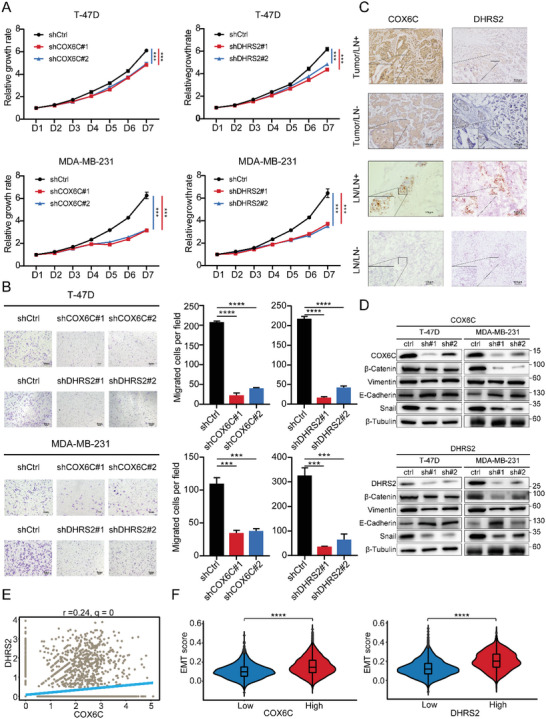
Downregulation of COX6C and DHRS2 inhibited breast cancer cells proliferation, migration, and EMT. A) Line plots showing significantly lower cell proliferation rates in T‐47D and MDA‐MB‐231 cells after knocking down COX6C and DHRS2. B) Bar plots showing downregulation of COX6C and DHRS2 significantly inhibited cell migration ability of T‐47D and MDA‐MB‐231 cells in trans‐well assay (right). Representative images randomly selected from T‐47D and MDA‐MB‐231 cells are shown (left). Scale bars = 1 mm. C) IHC images of COX6C and DHRS2 expressions in primary tumor with or without lymph node metastasis. Three independent experiments were performed and generated similar results. Scale bar = 100 µm. D) Western blot images showing the EMT signaling pathway was inactivated in shCOX6C and shDHRS2 group, compared with control of MDA‐MB‐231 and 4T1 cells. E) Scatter plots with a significant positive spearman correlation for the expression of COX6C and those of DHRS2. F) Violin spots of EMT score of the high expression and the low expression of COX6C and DHRS2 in epithelial cells. All *p*‐values in (A) and (B) were determined using an unpaired two‐sided Student's *t*‐test. All *p*‐values in (F) were determined using an unpaired two‐sided Wilcoxon rank‐sum test. Data presented as the mean ± SD. of *n* = 3. ns, *p* ≥ 0.05; *, *p* < 0.05; **, *p* < 0.01; ***, *p* < 0.001; ****, *p* < 0.0001. EMT, epithelial–mesenchymal transition. shCOX6C, COX6C knockdown. shDHRS2, DHRS2 knockdown.

To evaluate the role of COX6C and DHRS2 in breast cancer cells, we utilized short hairpin RNA (shRNA) to generate T‐47D, MDA‐MB‐231, and MCF‐7 cell lines with low COX6C or DHRS2 expression. Downregulation of COX6C and DHRS2 notably inhibited the proliferation of breast cancer cells (**Figure** **5**A and Figure S5A, Supporting Information). In the transwell migration assay, migrated cells transfected with shIL4I1 were remarkably decreased by 60% or more compared with control (Figure 5B and Figure S5B, Supporting Information), indicating high levels of COX6C and DHRS2 could facilitate breast cancer invasion. We next performed immunohistochemistry (IHC) staining on lymph nodes and primary tumors with positive or negative lymph node metastasis. We observed that the representative protein expression of COX6C and DHRS2 was positive in samples with lymph node metastasis (Figure 5C). Fisher's exact test showed the expression of both COX6C and DHRS2 are significantly related to the presence of lymph node metastasis (Tables S1 and S2, Supporting Information). Interestingly, the expression of E‐cadherin was upregulated while Snail was downregulated in COX6C knockdown (shCOX6C) and DHRS2 knockdown (shDHRS2) cells (Figure 5D and Figure S5C, Supporting Information), which suggested that COX6C and DHRS2 may promote cell migration through upregulating EMT‐related pathways. Moreover, lower levels of *β*‐Catenin in shCOX6C and shDHRS2 cells than in control indicated the downregulated WNT signaling pathway. Meanwhile, the analysis results based on scRNA‐seq dataset also displayed the positive correlation among COX6C, DHRS2, and the EMT pathway (Figure 5E,F). Taken together, these data demonstrated that the downregulation of COX6C and DHRS2 might inhabit cell proliferation, migration, and EMT in breast cancer.

### 2.6 Upregulation of OXPHOS Exhibited Associations with Lymph Node Status and Prognosis in Breast Cancer Patients

**Figure 6:**
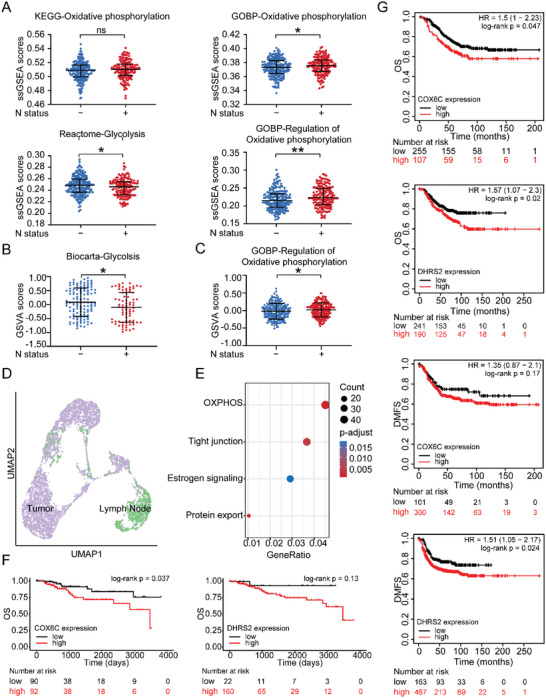
Clinical significance of OXPHOS in early disseminated breast cancer. A–C) Dot plots showing significantly higher level of OXPHOS and lower level of glycolysis in N status positive (N1‐3) breast cancer patients than those with N status negative (N0). Patients sequence data were from FUSCC (A), TCGA (B), and METABRIC (C). All *p*‐values were determined using an unpaired two‐sided Wilcoxon rank‐sum test. D) UMAP plot of 6350 cancer epithelial cells with tissue source. Each tissue source is shown in different color. E) Bubble plot of enriched KEGG pathways of cancer epithelial cell from lymph nodes compared to those from primary tumors. The intensity represents adjusted *p*‐value of each KEGG pathway. Dot size shows gene count for each KEGG pathway. Wilcoxon signed‐rank test was used to assess the difference. F) Kaplan–Meier curve illustrating higher COX6C and DHRS2 accompanied by poor OS in basal‐like breast cancer patients from TCGA dataset. G) Kaplan–Meier curve illustrating higher COX6C accompanied by poor OS and DMFS in HER2‐positive breast cancer while higher DHRS2 upregulation was associated with poor OS and DMFS in basal‐like breast cancer. Log‐rank test was used to assess the difference. ns, *p* ≥ 0.05; *, *p* < 0.05; **, *p* < 0.01; ***, *p* < 0.001; **** *p* < 0.0001. DMFS, distant metastasis‐free survival. GOBP, Gene Ontology Biological Process. KEGG, Kyoto Encyclopedia of Genes and Genomes. OS, overall survival. OXPHOS, oxidative phosphorylation. UMAP, Uniform Manifold Approximation and Projection.

To validate the association between OXPHOS pathway and breast cancer early dissemination, we applied single sample gene set enrichment analysis (ssGSEA) analysis in our FUSCC cohort and GSVA analysis in some patients from TCGA and METABRIC datasets.^[^ ^25^, ^26^, ^27^, ^28^ ^]^ Compared with patients without lymph nodes metastasis, those with lymph nodes metastasis demonstrated significantly higher scores in the OXPHOS pathway and lower scores in the glycolysis pathway (**Figure** **6**A–C), suggesting that the upregulation of OXPHOS might be related to lymph node metastasis and the metabolic switch from glycolysis to OXPHOS might take place.

To validate our findings in an external dataset, we selected the GSE167036 dataset as an external validation set,^[^ ^11^ ^]^ which includes the scRNA‐seq data of eight pairs of primary breast tumors and metastatic axillary lymph nodes. We performed low‐resolution Uniform Manifold Approximation and Projection (UMAP) clustering and identified cancer epithelial cells in eight patients. After cell annotation based on marker genes (Figure 6D), we conducted Kyoto Encyclopedia of Genes and Genomes (KEGG) analysis to identify pathways enriched in cancer epithelial cells from lymph nodes. As expected, OXPHOS, estrogen signaling, and tight junction pathways were enriched in the EDC cells (Figure 6E and Data S8, Supporting Information), which is consistent with our results (Figure 2D). Furthermore, COX6C upregulation was associated with poor overall survival (OS) in basal‐like breast cancer, while poor OS and distant metastasis‐free survival (DMFS) in HER2‐positive breast cancer. DHRS2 upregulation was associated with poor OS and DMFS in basal‐like breast cancer (Figure 6F,G). Collectively, these data validated that the OXPHOS pathway exhibited great value in predicting the risk of lymphatic metastasis and prognosis in breast cancer.

## 3 Discussion

In this study, we defined a specific cell cluster disseminated from the primary tumor site by performing scRNA‐seq in the primary breast cancer samples and paired metastatic lymph nodes. By integrating scRNA‐seq and ST, we characterized this EDC cluster from both spatial and temporal levels. During the evolvement of the early dissemination of breast cancer, the EDC cluster undergoes a transition between glycolysis and OXPHOS and tends to distribute along the tumor border. The previous study investigated the energy anabolism shift using scRNA‐seq in breast cancer patient‐derived xenografts models,^[^ ^21^ ^]^ our study has brought this finding more in‐depth by elaborating the bioenergetic state once the disseminated cells are seeded. Furthermore, our findings have significant clinical relevance since the sequencing is performed on paired clinical samples of primary breast cancer and metastatic lymph nodes.

Tumor cells go through a series of evolving events to become disseminated malignant cells. The evolvement occurs at multiple levels, including the genetic, epigenetic, and transcriptomic alterations, endowing the malignant cells with a more invasive phenotype.^[^ ^29^ ^]^ The malignant cells share characteristics such as genomic instability, cell death resistance, and altered metabolism. Based on these cancer hallmarks, they further acquired motility and invasion, plasticity, and experienced microenvironment modulation and EMT to become disseminated cells and eventually colonize at distant sites.^[^ ^30^ ^]^ The fast‐developing sequencing technology now allows us to look deeper at the transitions during this process. This study focused on the metabolism evolvement in the early disseminated breast cancer cells. The metabolic change in cancer cells plays an important role in regulating tumor metastasis,^[^ ^31^ ^]^ and the role of OXPHOS and glycolysis in tumor metastasis remains debatable. Increased OXPHOS was utilized in circulating cancer cells in breast cancer mouse model, where peroxisome proliferator‐activated receptor gamma, coactivator 1 alpha (PGC‐1*α*) promoted distant metastases via enhancing this pathway.^[^ ^32^ ^]^ The OXPHOS pathway promoted cellular invasion, possibly due to the increased mitochondrial superoxide from overactive mitochondrial metabolism.^[^ ^33^ ^]^ However, another study showed that OXPHOS negatively correlates with EMT, and the downregulation of genes in this pathway was noticed in patients with high survival rates.^[^ ^34^ ^]^ Inhibition of LDHA, the key enzyme of glycolysis, suppressed tumor migration in multiple cancer types.^[^ ^35^, ^36^, ^37^ ^]^ These controversial results are possibly due to the dynamic metabolic changes during metastasis; the fluctuating levels of OXPHOS and glycolysis might interfere with the consistency of the results of studies. Our study suggested that the upregulation of the OXPHOS is a temporal phase in the process of tumor dissemination, and once colonized, the disseminated group exhibits a more malignant property with upregulated glycolysis.

Our study localized EDC clusters at the tumor leading edge, in other words, early disseminated tumor cells accumulate along the tumor‐host interface. The tumor‐host microenvironment allows extensive cellular communication between tumor cells and mesenchymal stromal components.^[^ ^38^ ^]^ In squamous cell carcinoma, a tumor‐specific cluster of cells localized on the leading edge of tumor, where they interact with multiple cell types and exhibited invasive phenotype.^[^ ^39^ ^]^ Cellular interactions are also crucial in breast cancer. Neighboring cells such as fibroblasts and immune cells facilitate dissemination.^[^ ^40^ ^]^ In this study, we applied CellChat analysis on scRNA‐seq and identified that the MIF pathway is highly activated between disseminated tumor cells and immune cells. MIF is a cytokine that binds and activates receptors CD74/CD44, CXCR2, CXCR4, and CXCR7, which has been reported to play an essential role in malignant diseases and as a novel therapeutic approach against triple‐negative breast cancer.^[^ ^41^, ^42^ ^]^ Further studies are still needed to clarify the relationship between MIF and metastasis.

This study still has several limitations. First is the high spatial heterogeneity among samples, which makes it hard to obtain shared characteristics. The second is the limited sample numbers. We performed scRNA‐seq and ST in four patients, limiting our conclusion's generalization. However, Liu et al. have characterized the microenvironment of primary tumors and paired metastatic lymph nodes by scRNA‐seq.^[^ ^11^ ^]^ In this study, we set their dataset as an external validation set to further confirm the metabolic traits of the metastatic cancer cells.

We used two ways to combine scRNA‐seq and ST to make the result more convincing. Last but not least, early dissemination could occur either in a hematogenous way or via lymph way. However, early detection of hematogenous metastasis is difficult and unstable. Here we only focus on dissemination in the lymph ways, and further research on early hematogenous dissemination is needed.

Our results uncovered the metabolism alterations in disseminated cells and revealed the dynamic evolvement of breast cancer cells during early dissemination. Our results warrant further investigation of the breast cancer early dissemination mechanism and have potential prognostic and therapeutic value.

## 4 Experimental Section

### Ethical Statement

This study was reviewed and approved by the Institutional Review Board of FUSCC. Each patient provided written informed consent before sample collection.

### Human Specimens

This study included four patients who underwent surgery at FUSCC. In total, eight fresh surgical specimens (four primary tumors and four paired metastatic lymph nodes) were sequenced and incorporated in further analyses. The metastasis of lymph nodes was confirmed by pathologists through both cytological detections during the surgery and the paraffin section after surgery. Clinical information, including demographics and tumor clinicopathologic characteristics, are summarized in Data S1, Supporting Information (the staging was performed according to the American Joint Committee on Cancer system 8 at the time of diagnosis).

### Cell Lines and Cell Culture

The human breast cancer cell line MDA‐MB‐231 and murine 4T1 were purchased from ATCC. MDA‐MB‐231 and T‐47D breast cancer cells were cultured in Roswell Park Memorial Institute (RPMI) 1640 medium supplemented with 5% fetal bovine serum (FBS, Gibco) and 1% pen–strep antibiotic (Beyotime). MCF‐7 breast cancer cells were cultured in Eagle's Minimal Essential Medium supplemented with 10%FBS and 1% pen–strep antibiotic. HEK293T cells were cultured in Dulbecco's Modified Eagle Medium (Thermo Fisher) supplemented with 10% FBS and 1% pen–strep antibiotic. All of the cell lines were tested and authenticated. All cells were cultured at 37 °C in a humidified atmosphere containing 5% CO_2_. The cell lines were Mycoplasma‐free and authenticated by PCR analysis monthly.

### Tumor Sample Handling and Dissociation to a Single‐Cell Suspension for scRNA‐Seq

Tumor and lymph node samples were resected by an experienced physician to ensure that the tumor samples contained neoplastic, stromal, and para‐cancerous tissue and the lymph node samples consisted of neoplastic and lymphoid tissue with complete cross section. Fresh samples were washed two times with precooling 0.04% bovine serum albumin (BSA, MACS) in RPMI 1640 medium and evenly divided into two parts for scRNA‐seq and ST. Each sample for scRNA‐seq was cut into ≈1 mm^3^ pieces and enzymatically digested with 10 mL digestion medium containing 0.2% collagenase type IV (Gibco), 0.05% hyaluronidase type I‐S (Sigma‐Aldrich), and 0.002% DNase I (Applichem). After 45 min of digestion at 37 °C in a shaking water bath, the enzymatic hydrolysate was filtered through a 40‐µm pore size nylon mesh and centrifuged at 1500 rpm for 5 min to obtain a single‐cell suspension. Next, erythrocytes were lysed using a red blood cell lysis solution (MACS) for 5 min. The cell suspension was centrifuged at 1500 rpm for 5 min. After removing the supernatant, the cell pellet was washed twice with RPMI 1640 with 0.04% BSA and then re‐suspended in the sorting buffer (PBS supplemented with 0.04% FBS). Cell concentration and viability were assessed by Luna cell counter.

### scRNA‐Seq and Reads Processing

The cell concentration of fresh cell suspension for each sample was adjusted to 700–1200 cells/µL. Then the cell suspension was subjected to Chromium Next GEM Single Cell 3ʹ Reagent Kits v3.1 (10x Genomics, Pleasanton, CA) for library preparation according to the standard protocols. The single cell libraries were sequenced on Illumina NovaSeq 6000 Systems using paired‐end sequencing (150 bp). The Cell Ranger software pipeline (version 3.1.0) provided by 10x Genomics was used to demultiplex cellular barcodes, map reads to the genome and transcriptome using the STAR aligner, and down‐sample reads as required to generate normalized aggregate data across samples, producing a matrix of gene counts versus cells.

### Quality Control and Batch Effect Correction of scRNA‐Seq Data

The UMI count matrix was processed using the R package Seurat (version 3.1.1).^[^ ^15^ ^]^ To remove low‐quality cells and likely multiplet captures, which was a major concern in microdroplet‐based experiments, the following criteria were applied to filter out cells with UMI/gene numbers out of the limit of mean value +/− twofolds of standard deviations assuming a Gaussian distribution of each cells' UMI/gene numbers. Following visual inspection of the distribution of cells by the fraction of mitochondrial genes expressed, low‐quality cells where >10% of the counts belonged to mitochondrial genes were discarded . After applying these quality control criteria, 65 968 single cells were included in downstream analyses. Library size normalization was performed with NormalizeData() function in Seurat to obtain the normalized count. Specifically, the global‐scaling normalization method “LogNormalize” normalized the gene expression measurements for each cell by the total expression, multiplied by a scaling factor (10 000 by default), and the results were log‐transformed. After that, the normalized expression profiles of all samples were merged together using the merge() function in R v3.6.3. To remove the batch effects in single cell RNA sequencing data, the mutual nearest neighbors presented by Haghverdi et al. was performed with the R package batchelor.^[^ ^43^ ^]^ The top 5000 highly variable genes (HVGs) of the merged dataset identified by the FindVariableFeatures() function were utilized as input for batch effect correction. Finally, the scaled and batch effect‐corrected expression profiles of all samples were obtained for downstream analyses.

### Unsupervised Clustering and Dimensional Reduction

The top principal components (PCs) were computed based on the gene expression profiles of the top 5000 HVGs after batch effect correction. The PCElbowPlot() function in Seurat was utilized to select the optimal number of PCs for further analysis as recommended by Seurat. The FindNeighbors() and FindClusters() functions in Seurat were both applied for cell clustering. The RunTSNE() and RunUMAP() function were both performed for visualization when appropriate. In the first round of “low‐resolution” clustering, 15 clusters were found from 65 968 single cells. To resolve the high heterogeneity in tumor tissue, the second round of “high‐resolution” clustering was conducted to identify the finer clusters within in epithelial cell. Procedures of the second round of clustering were identical to the first one, both starting from the computation of PCs and then clustering cells with the optimal resolution obtained from the “clustering tree” method to seek the identity for each cluster.

### Identification of Marker Genes for Cell Clusters and Cell Type Annotation

The marker genes in each cluster were identified through the FindAllMarkers() function in Seurat. Significant levels of these signature genes were determined using the bimod test. For a given cluster, FindAllMarkers identified positive markers compared with all other cells. Then, the R package SingleR,^[^ ^44^ ^]^ with the reference transcriptomic datasets “Human Primary Cell Atlas”^[^ ^45^ ^]^ was used to infer the cell of origin of each of the single cells independently and identify cell types. In the first round of “low‐resolution” clustering, epithelial cells (EPCAM, CDH1, KRT8, KRT18), endothelial cells (PECAM1, CDH5, CD34, VWF), B cells (CD79A, CD79B, CD19, IGHD), T/natural killer (NK) cells (CD3D, CD3G, FCGR3A, NKG7), and fibroblasts (COL1A1, COL1A2, COL3A1, ACTA2) were identified.

### Single‐Cell Copy‐Number Variation Evaluation

The CNV evaluation of each cell for each region on the chromosome was conducted by infercnv R package (version 1.0.4) (https://github.com/broadinstitute/inferCNV)^[^ ^18^ ^]^ based on the amount of gene expression in the single‐cell transcriptome data. The CNV levels of epithelial cells were calculated and other cells were applied as the reference. The genes were sorted by chromosomal location, and the average gene expression was calculated using 101 genes as the sliding window size, and then normal cell expression was used as a control to generate the final CNV result file after denoising. The parameters of inferCNV analysis included “denoise,” default hidden Markov model settings, and a value of 0.1 for ″cutoff." To reduce the false‐positive CNV calls, the default Bayesian latent mixture model was implemented to identify the posterior probabilities of the CNV alterations in each cell, with the default value of 0.5 as the threshold. The phylogenetic tree of tumor evolution was draft using Uphyloplot2^[^ ^46^ ^]^ software based on the CNV data, and the phylogenetic tree of each sample was classified according to tumor location and mutation status.

### Identification of Signature Genes among Cell Clusters

Differentially expressed genes (DEGs) were identified using the FindMarkers() function of Seurat package. Significance levels of these signature genes were determined using the Wilcoxon rank‐sum test along with Bonferroni correction. *p*‐value <0.05 and |log2foldchange| >0.5 was set as the threshold for significantly differential expression.

### Inference of Cell Fate by Trajectory Analysis

The trajectory analysis was performed using the Monocle2 package (version 2.9.0)^[^ ^47^ ^]^ to reveal the cell‐state transitions. The ordering genes in the trajectory analysis were determined according to each gene's expression (mean expression > 1) and variance level (dispersion_empirical > 2 × dispersion_fit) as recommended by Monocle2. The importCDS() function converted the Seurat object to a CellDataSet object. Then the differentialGeneTest() function was applied to filter out the genes used to sort cells (ordering gene, qval < 0.01). The reduceDimension() function in Monocle2 was applied to reduce the dimensions. All functions were set with default settings. As the normal ductal epithelial cells were separated and clearly defined in this study, these cells were set as the root state and performed the “orderCell” function in Monocle2. The DEGs changed along with the pseudotime were identified using the differentialGeneTest() function in Monocle2.

### Tissue Processing and Visium Data Generation

Breast cancer tissues were gently washed with cold 1× PBS (Gibco) and evenly divided for preparing a single‐cell suspension (see above) and ST. The fresh frozen tumor tissues for ST were stored at −80 °C and embedded in OCT (Sakura, Alphen aan den Rijn, Netherlands) before cryosectioning for RNA extraction. Each tumor cryosection was cut with thicknesses of 10 µm in the cryostat (Leica CM1950, Germany) and mounted onto ST microarrays. For processing, the tissue was dehydrated with isopropanol for 1 min and staining with H&E.

Bright‐field images were obtained by a whole slide scanner (Pannoramic MIDI FL, 3DHISTECH) at 20× resolution. H&E‐stained sections of each sample were carefully reviewed by two experienced pathologists to confirm the pathology, and were then manually annotated by a trained pathologist to identify tumor, stromal, and normal ductal region.

### ST Barcoded Microarray Slide Information

Library preparation slides used were purchased from the ST team (https://www.spatialtranscriptomics.com). Each of the spots printed onto the array was 55 µm in diameter and 100 µm from center to center, covering an area of 6.5 × 6.5 mm^2^. Each slide included four capture zones. Each capture zone had ≈5000 unique gene expression spots.

### Tissue Permeabilization, cDNA Synthesis, Library Construction, and Sequencing

Tissue sections were subjected to Visium Spatial Gene Expression Reagent Kits (10× Genomics, Pleasanton, CA) for library preparation according to the standard protocols. First, fixed and stained tissue sections were permeabilized with 70 µL permeabilization enzyme at 37 °C for 18 min. Then each well was washed with 100 µL SSC and 75 µL reverse transcription Master Mix containing reverse transcription reagents were added for cDNA Synthesis. After incubation with reagents, full‐length cDNAs with spatial barcodes generated from polyadenylated mRNAs were obtained . At the end of first‐strand synthesis, RT Master Mix was removed from the wells with the subsequent addition of 75 µL 0.08 m KOH and incubated for 5 min at room temperature. KOH was then removed from wells, and the residuals were washed with 100 µL EB buffer. In all, 75 µL second strand mix was added to each well for second‐strand synthesis. Finally, spatial barcodes were encoded and the full‐length cDNA was amplified by PCR to obtain a sufficient amount for library construction. Libraries were prepared according to the Visium Spatial Gene Expression User Guide (CG000239, Rev B, 10x Genomics). The constructed library was sequenced on Illumina NovaSeq 6000 Systems with a sequencing depth of at least 100 000 reads per spot with a pair‐end 150 bp reading strategy.

### ST Library Sequence Alignment, Annotation, and Data Analysis

The Space Ranger Pipeline (version 1.0.0, 10X Genomics, Pleasanton, CA)^[^ ^48^ ^]^ was used to conduct data quality statistical analyses of the ST sequencing data. Reads were mapped to the human GRCh38 genome assembly and aligned using STAR. Spots with more than 10% mitochondrial genes or fewer than 200 detected gene counts were discarded. The UMI count matrix was processed using the R package Seurat (version 3.1.1). First, the data were normalized with sctransform^[^ ^49^ ^]^ in order to account for variance in sequencing depth across data points, detect high‐variance features, and store the data in the SCT assay.

Top variable genes across single cells were identified using the method described by Macosko et al.^[^ ^50^ ^]^ Briefly, the average expression and dispersion were calculated for each gene, genes were subsequently placed into x bins based on the expression. Principal component analysis was performed to reduce the dimensionality on the log‐transformed gene‐barcode matrices of top variable genes. Cells were clustered based on a graph‐based clustering approach, and were visualized in 2D using tSNE. Likelihood ratio test that simultaneously tests for changes in mean expression and in the percentage of expressed cells was used to identify significantly DEGs between clusters.

To identify molecular features that correlate with spatial location within a tissue, Seurat was used to perform differential expression based on pre‐annotated anatomical regions within the tissue, which may be determined either from unsupervised clustering or prior knowledge. The BMS score was calculated for each ST spot using the AddModuleScore() function with default parameters in Seurat with the top 25–100 significantly DEGs between EDCs and other epithelial cells in four tumor samples. To deconvolute cell types within scRNA‐seq, first the cell number of each cell type was down‐sampled to 100 in the corresponding scRNA‐seq data, and then SPOTlight^[^ ^23^ ^]^ was applied to deconvolute and seven cell types were mapped to ST section. The probability was set lower than 0.2 of each cell type as noise, then chose the highest probability out of all cell type as the identified cell type.

### Determination of Cell Type Enrichment/Depletion by MIA

MIA was an analytical method that integrated single‐cell expression profiles with spatial transcriptome data.^[^ ^15^ ^]^ This analysis was proceeded by first delineating sets of cell type‐specific and tissue region‐specific genes and then determining whether their overlap was higher (enrichment) or lower (depletion) than expected by chance. After obtaining two kinds of gene sets, the significance of the overlap between ST genes and cell type marker genes was queried using the hypergeometric cumulative distribution, with all genes as the background to compute the *p*‐value. In parallel, test was done for cell type depletion by computing −log10(1 − *P*).

### Cell–Cell Communication Analysis

To infer the potential intercellular communication network between clusters, CellChat R package (version 1.1.3)^[^ ^22^ ^]^ was used to quantitatively measure networks through the law of mass action based on the average expression values of a ligand by one cell group and that of a receptor by another cell group, as well as their cofactors. The normalized expression matrix was imported and a cellchat object was created with the createCellChat() function. After preprocessing data with dentifyOverExpressedGenes(), identifyOverExpressedInteraction(), and projectData(), the computeCommunProb, filterCommunication (min.cells = 10), and computeCommunProbPathway functions were used to calculate potential ligands–receptor interactions. Finally, using the “aggregateNet” function in CellChat, the aggregated cell–cell communication network was calculated.

### KEGG Enrichment Analysis, Hallmark Pathway Enrichment Analysis, GSVA, and ssGSEA of DEGs

To determine the biological and molecular functional processes of the prioritized gene list as well as their significant enriched pathways, the KEGG enrichment analysis and Hallmark pathway enrichment analysis were performed by clusterProfiler (Version 3.6.0) with *p* <0.05. To find which functional pathways work in specific cell clusters, GSVA package (version 1.30.0)^[^ ^51^ ^]^ was used to perform gene set enrichment analyses based on H dataset (version 7.2) from MsigDB (http://www.gsea‐msigdb.org/gsea/msigdb/) and calculated scores for pathway activity values in each cell. Finally, LIMMA package (version 3.38.3) was used to calculate the difference in signaling pathway activity between different groups. Pathways with adjusted *p*‐value >0.05 were not considered for further analysis. The upregulated and downregulated pathways were visualized by bar plot. Also, ssGSEA was used that define an enrichment score to represent the degree of absolute enrichment of a gene set in each sample, including KEGG, Gene Ontology Biological Process, and Reactome pathways. Normalized enrichment scores could be calculated for OXPHOS or glycolysis pathway. The ssGSEA analysis was performed in R package GSVA. *p*‐value <0.05 was considered as statistical significance.

### Plasmid/shRNA Construction and Virus Infection

Human COX6C and DHRS2 were amplified with the reverse‐transcribed cDNA from MDA‐MB‐468, T‐47D, and MCF‐7 cell lines and cloned into the lentiviral vector pSIN‐Flag (puromycin‐resistant; Addgene), and the authenticity was verified by sequencing, respectively. shRNA sequences of COX6C and DHRS2 were purchased from Sigma‐Aldrich and cloned into the lentiviral vector pLKO.1 (Addgene). A highly efficient lentiviral system was used to generate the viruses. The cell lines were infected with the lentiviruses, and stable cell lines were established. The lentiviral transfection efficiency was more than 90% in all cell lines.

### Western Blot Analysis

Total protein was extracted using RIPA buffer (Thermo Fisher Scientific, Waltham, MA, USA) and detected utilizing BCA assay (Thermo Fisher Scientific, Waltham, MA, USA). 30 µg of protein per sample was separated by SDS‐PAGE, and then transferred onto PVDF membrane (Gene Molecular Biotech, Inc., Shanghai, China). After obturated with 5% milk for 2 h at room temperature, the membrane was incubated overnight at 4 °C with primary antibodies as follow: COX6C (2 µg mL^−1^, Abcam, ab110267), DHRS2 (0.1 µg mL^−1^, Abcam, ab220483), *β*‐Catenin (1:5000, Abcam, ab32572), Vimentin (1:1000, Abcam, ab92547), E‐cadherin (1:10 000, Abcam, ab40772), Snail (1:1000, Abcam, ab216347), and *β*‐tubulin (1:1000, Abcam, ab179511). Afterward, the membrane was incubated with HRP‐conjugated goat anti‐rabbit IgG secondary antibodies (1:7500, Abcam, ab205718) or HRP‐conjugated goat anti‐mouse IgG secondary antibodies (1:7500, Abcam, ab205719) for 1 h at room temperature, the expression level was measured with an ECL kit (Roche Diagnostics, Basel, Switzerland) by Western blot imaging system.

### Cell Proliferation Assays

1 × 10^3^ cells were incubated in 96‐well plates. Then, 10 µL Cell Counting Kit‐8 (Dojindo, Rockville, MD, USA) solution was added to each well and incubated for 2 h to evaluate cell proliferation. The absorbance of each well was measured at OD450 with a Tecan Infinite M1000 PRO (Tecan, Switzerland) from days 1 to 7.

### Transwell Assays

The transwell migration and invasion assays were conducted with Corning Transwell Inserts (8.0 µm). For the transwell migration assay, 1.5 × 10^4^ transfected cells suspended in 50 µL serum‐free medium were placed in the upper chamber and 600 µL medium (10% FBS) was filled in the lower compartment. The cells were incubated at 37 °C for 24 h. The successfully translocated cells were fixed with 4% paraformaldehyde and stained with 0.1% crystal violet, and counted in four randomly chosen fields (200×) under a microscope. For the transwell invasion assay, 1.2 × 10^5^ cells were seeded on transwell coated with 50 µL Matrigel (dilution of 1:4 with 0.2% BSA). It is worth noting that Matrigel was used to coat membranes for 12 h at 37 °C before invasion assays. The culture condition was the same as the transwell migration assay. The cells on the lower surface were fixed, stained, and photographed microscopically after 48 h.

### Immunohistochemical Staining of Formalin‐Fixed, Paraffin‐Embedded Tissue

Formalin‐fixed, paraffin‐embedded tumor tissues of two additional cases were separately cut into 5‐µm sections and mounted on glass slides. The slides were baked at 65 °C overnight. After deparaffinization and hydration, these slides were boiled in citrate buffer at 100 °C for 15 min. Subsequently, a 3% H2O2 solution was used to block endogenous peroxidase activities for 20 min. To prevent nonspecific antibody binding, the slides were incubated with 5% normal goat serum for 1 h at room temperature. Then these slides were incubated at 4 °C overnight with anti‐COX6C and anti‐DHRS2 primary antibody (Abcam, ab110267 and ab220483), 1:100, which was validated for IHC by the manufacturer on HeLa cells and RT‐4 cells, respectively. Following washes with TBST for three times, the slides were then incubated with HRP‐conjugated goat anti‐rabbit/mouse secondary antibody (GeneTech, GK500705) for 1 h at room temperature. Sections were stained by DAB and then counterstained with hematoxylin according to the manufacturer's instructions.

### Quantification and Statistical Analysis

All the statistical analyses were performed using R (version 3.6.1) and GraphPad Prism 8.0. The assay was repeated at least three times and the data were presented as mean ± standard deviation. Student's *t*‐test, bimod test, Fisher's exact test, Wilcoxon rank‐sum test, likelihood ratio test, spearman correlation analysis, and log‐rank test were utilized in this study. *p*‐values of less than 0.05 were considered statistically significant (ns, *p* ≥ 0.05; *, *p* < 0.05; **, *p* < 0.01; ***, *p* < 0.001; ****, *p* < 0.0001).

## Conflict of Interest

The authors declare no conflict of interest.

## Author Contributions

Y.‐M.L., J.‐Y.G., and Y.‐F.C. contributed equally to this work. Conceptualization: K.‐D.Y. and Z.‐M.S. Methodology: Y.‐M.L., J.‐Y.G., L.C., and Y.‐W.C. Investigation: D.M., Y.‐Y.C., and C.‐C.L. Visualization: Y.‐M.L. and J.‐Y.G. Supervision: K.‐D.Y., Z.‐M.S., Y.‐Y.X., and C.‐C.L. Writing—original draft: Y.‐M.L. and Y.‐F.C. Writing—review & editing: K.‐D.Y. and Z.‐M.S. All authors read and approved the final manuscript.

## Supporting information

Supporting Information

Supplemental Dataset 1

Supplemental Dataset 2

Supplemental Dataset 3

Supplemental Dataset 4

Supplemental Dataset 5

Supplemental Dataset 6

Supplemental Dataset 7

Supplemental Dataset 8

## Data Availability

Data are available in a public, open access repository. All raw scRNA‐seq and scTCR‐seq will be deposited to GEO shortly, and the accession number will be provided when available. Processed scRNA‐seq data from the external dataset are available through the Gene Expression Omnibus under accession number GSE176078. FUSCC TNBC sequence data were available in the NCBI Gene Expression Omnibus (OncoScan array; GSE118527) and Sequence Read Archive (whole‐exome sequencing and RNA‐seq; SRP157974). TCGA and MATABRIC TNBC sequence data were available in the cBioPortal (https://www.cbioportal.org/). Kaplan–Meier survival plots were generated online with the Kaplan–Meier plotter database (https://kmplot.com/analysis/), and hazard ratios with 95% confidence intervals and log‐rank *p*‐values were calculated.
