# Supplementary material for: Combined Single‐Cell and Spatial Transcriptomics Reveal the Metabolic Evolvement of Breast Cancer during Early Dissemination
Source: Adv Sci (Weinh). 2023 Jan 3;10(6):2205395. doi: 10.1002/advs.202205395 (PMC9951304; doi:10.1002/advs.202205395)
Supplement: Supplementary file 1 — Supporting Information [file ADVS-10-2205395-s007.pdf]

Supporting Information

Combined single-cell and spatial transcriptomics reveal the metabolic evolvement of breast cancer during early dissemination

Yi-Ming Liu[1,2,#], Jing-Yu Ge[2,#], Yu-Fei Chen[2#], Tong Liu[4], Lie Chen[2], Cui-Cui Liu[1], Ding Ma[1], Yi-Yu Chen[2], Yu-Wen Cai[2], Ying-Ying Xu[5], Zhi-Ming Shao[1,3], Ke-Da Yu[1,2,3,]*
[#]These authors contributed equally to this work.
**Corresponding author*

This supporting information includes:

Figures. S1 to S5

Tables S1 to S2

Legends for data S1 to S8

Other Supplementary Materials for this manuscript include the following:

Data S1 to S8

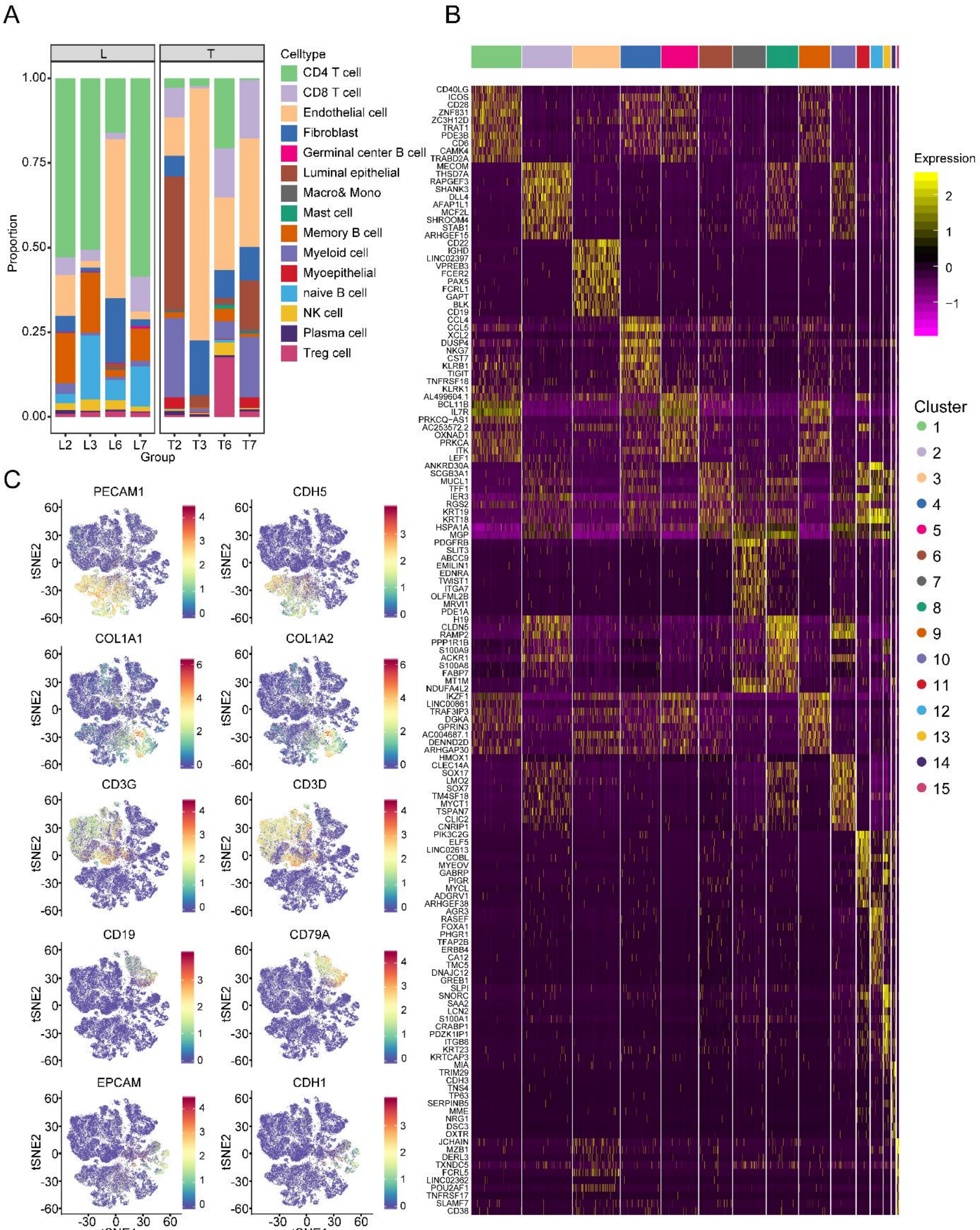

Figure S1. Cell types identified by mark genes of sc-RNA seq clusters. (A) Bar plots shows the relative proportion of cell types across four primary tumors and paired lymph nodes. Each cell type is shown in different color. **(B)** Heatmap showing expression of top 10 marker genes from each sc-RNA seq cluster. The intensity represents expression of top 10

marker genes in sc-RNA seq datastet. **(C)** Expression levels of selected known marker genes across 65,968 unsorted cells illustrated in t-SNE plots from both primary cancer and lymph node tissue in breast cancer patients.

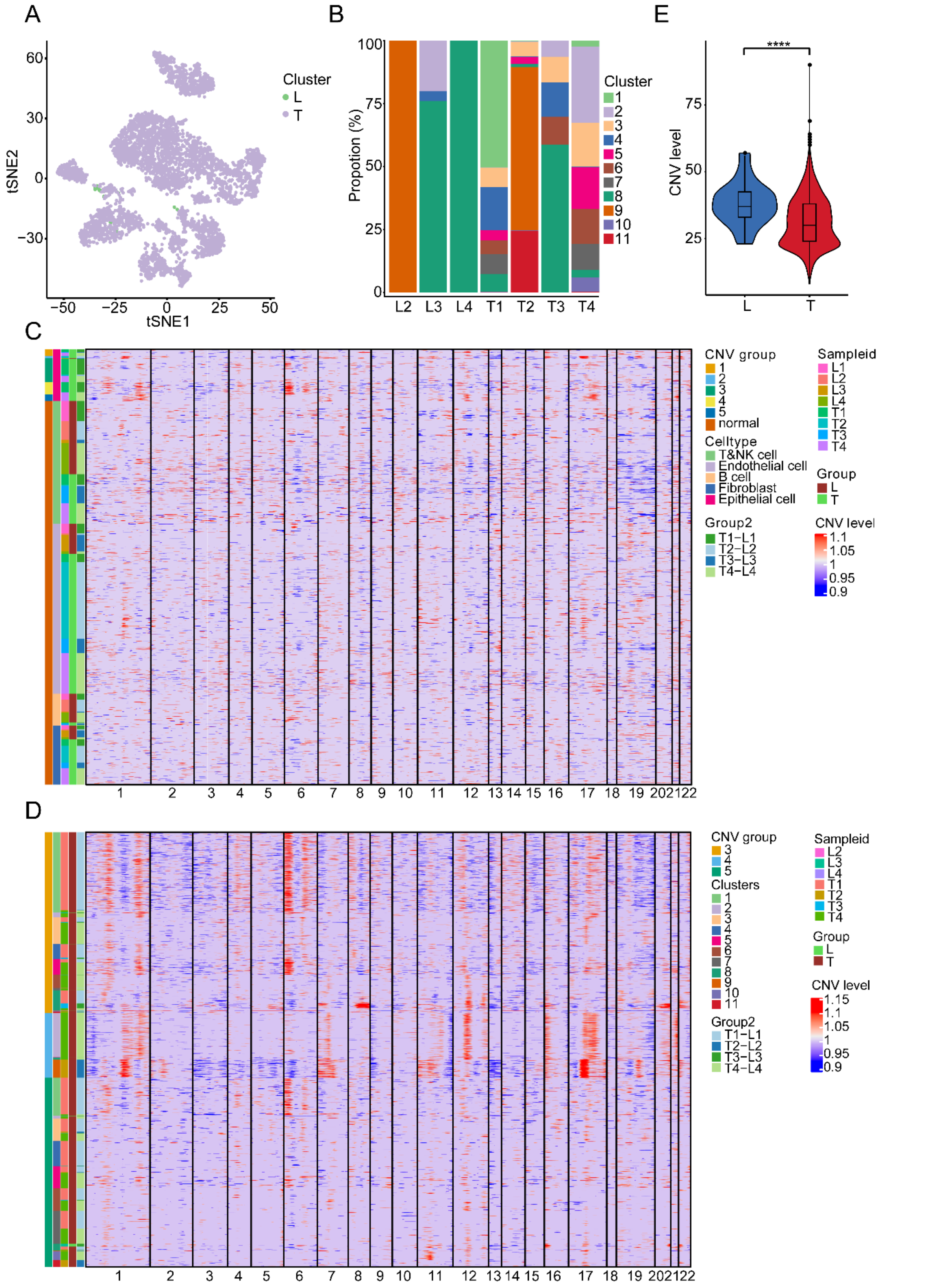

Figure S2. Malignant epithelial cells identified by CNV levels. (A) t-SNE plot of 5,739 cells from 4 pairs of tumor and lymph node tissue. Each tissue source is shown in different

color. **(B)** Bar plot showing the proportion of epithelial cell clusters in each lymph node and tumor sample. Each cluster is shown in different color. **(C)** InferCNV profiles of all cells from four paired primary breast tumors and metastatic lymph nodes. **(D)** InferCNV profiles of epithelial cells from four paired primary breast tumors and metastatic lymph nodes. **(E)** Violin spot of CNV levels of the epithelial cells in primary tumors and lymph nodes.

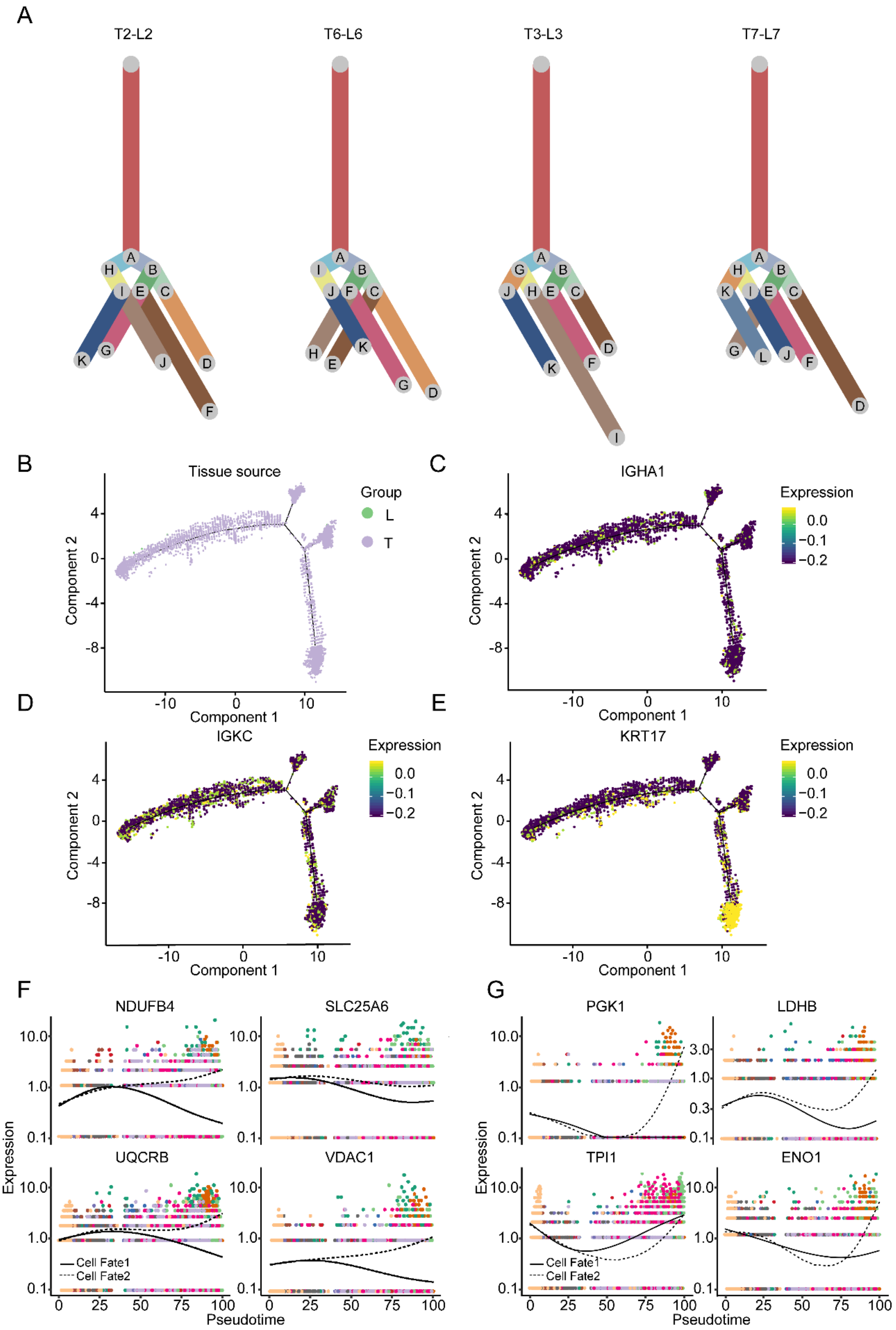

Figure S3. Evolution of epithelial cells from primary tumor to lymph node. (A) Clonality trees of epithelial cells of each sample from primary tumors to lymph nodes. The branches are

scaled according to percentage of cells in the calculated subclone containing the corresponding CNVs. (**B**) Potential trajectory of all epithelial cells showing the tissue source of epithelial cells. Each tissue source is shown in different color. (**C-E**) Potential trajectory of epithelial cells showing the expression of transformation along this trajectory, including IGHA1 (**C**), IGKC (**D**), and KRT17 (**E**). The intensity represents the normalized expression of each gene. **(F-G)** Dot plots of dynamic expression of key genes representing oxidative phosphorylation **(F)** and glycolysis **(G)** along two cell fates.

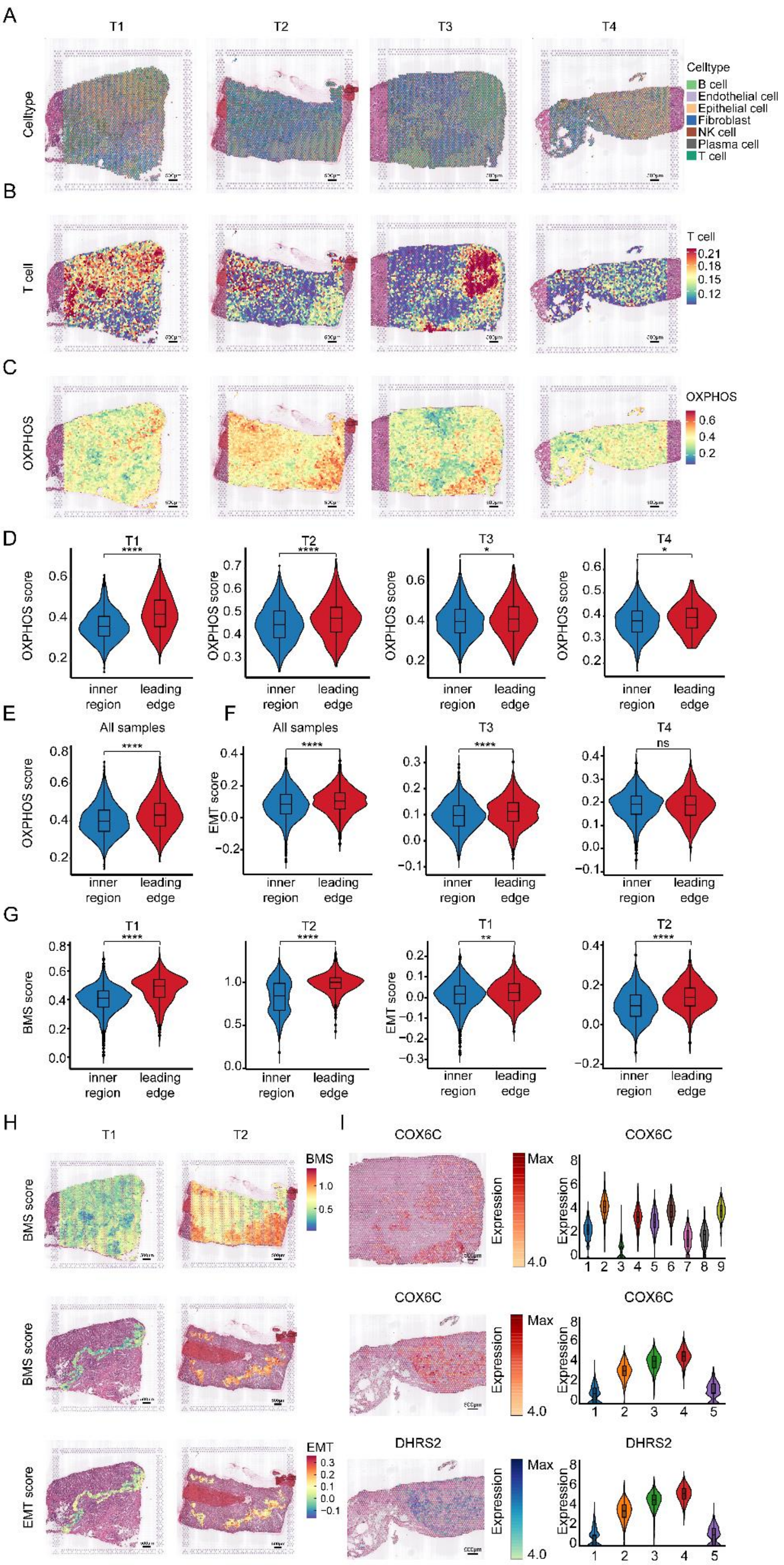

Figure S4. Features of spatial transcriptomics dataset from primary tumors. (**A**) All ST spots with cell types proportion inferred by SPOTlight. Scale bar, 500 µm. Each celltype is

shown in different color. (**B**) The proportion of T cell of all ST spots in T1-T4 tissue sections. The intensity represents proportion of each ST spot. Scale bar, 500 µm. (**C**) All ST spots of OXPHOS scores in T1-T4 tissue sections. The intensity represents score of each ST spot. Scale bar, 500 µm. (**D-E**) Violin spots of OXPHOS score of the tumor inner region and the tumor leading edge in T1-T4 and 4 samples together. **(F)** Violin spots of EMT score of the tumor inner region and the tumor leading edge in T3, T4 and 4 samples together. **(H)** All ST spots of BMS score and the tumor leading edge ST spots of BMS and EMT score in T1 and T2 tissue sections. The intensity represents score of each ST spot. Scale bar, 500 µm. (**I**) Violin spots of BMS and EMT score of the tumor inner region and the tumor leading edge in T3 and T4 sample, respectively. All *p* values were determined using an unpaired two-sided Wilcoxon rank-sum test. ns, $p \geq 0.05$, *, $p < 0.05$; **, $p < 0.01$; ***, $p < 0.001$, **** $p < 0.0001$

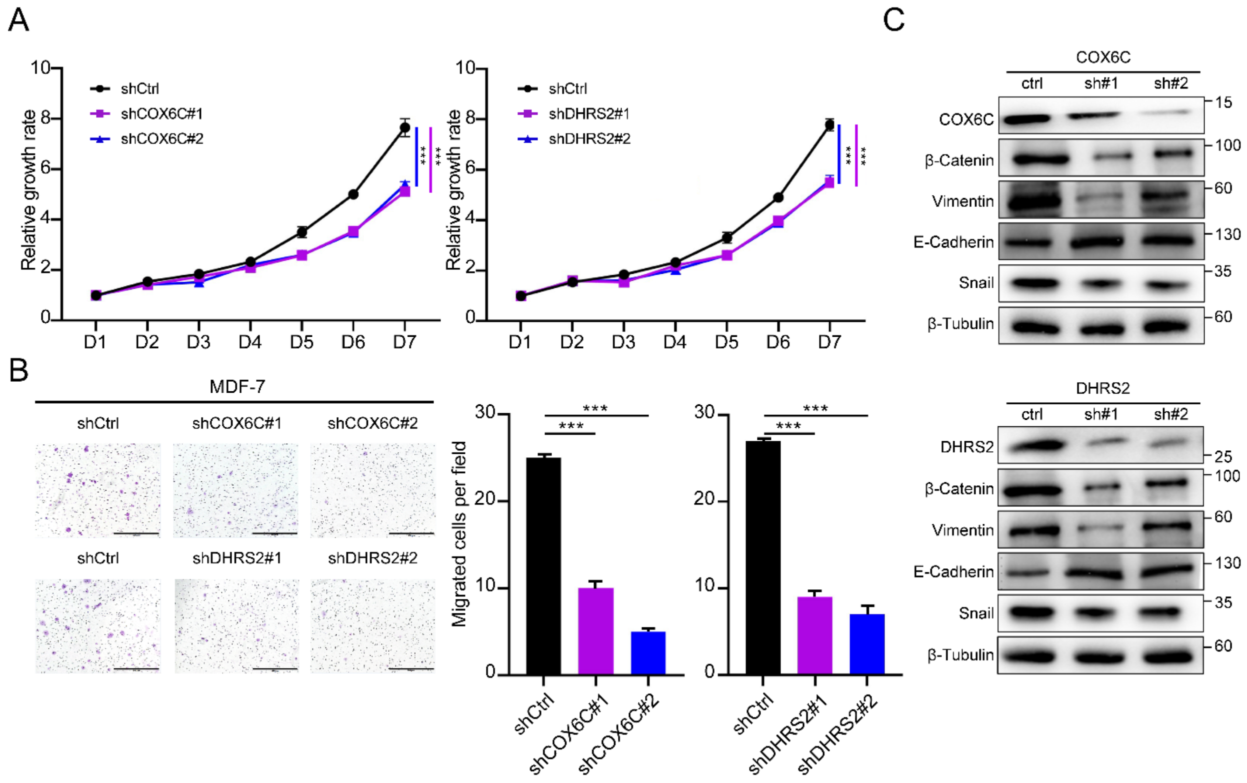

Figure S5. Knocking down COX6C and DHRS2 in MCF-7 inhibited proliferation, migration and epithelial-mesenchymal transition. (A) Line plots showing significantly lower cell proliferation rates in MCF-7 cells after knocking down COX6C and DHRS2. **(B)** Bar plots showing downregulation of COX6C and DHRS2 significantly inhibited cell migration ability of MCF-7 cells in trans-well assay (right). Representative images randomly selected from MCF-7 cells are shown (left). Scale bars, 500 µm. **(C)** Western blot images showing the EMT signaling pathway was inactivated in shCOX6C and shDHRS2 group, compared with control of MCF-7 cells.All *p* values were determined using an unpaired two-sided Student's t-test. Data presented as the mean ± s.d. of n = 3. ns, $p \geq 0.05$, *, $p < 0.05$; **, $p < 0.01$; ***, $p < 0.001$, **** $p < 0.0001$.

Table S1. IHC scores of COX6C expressions in primary tumor with or without lymph node metastasis. *p* values were determined using Fisher's exact test.

IHC Score (COX6C)	LN status +	LN status -	Total	*p* value
+	8	1	9	
-	0	4	4	0.007
Total	8	5	13	

Table S2. IHC scores of DHRS2 expressions in primary tumor with or without lymph node metastasis. *p* values were determined using Fisher's exact test.

IHC Score (DHRS2)	LN status +	LN status -	Total	*p* value
+	7	1	8	
-	1	4	5	0.032
Total	8	5	13	

Captions for data S1-S8

Data S1. Demographics and clinicopathological characteristics of the study population

Data S2. Cell type annotation of each cell

Data S3. Enriched hallmark pathways in EDC clusters

Data S4. Intercellular interaction between EDCs and immune cells in tumor and paired lymph node

Data S5. The proportion of each cell type in all ST spots

Data S6. Top 65 genes for BMS score in Spatial transcriptome dataset

Data S7. Enriched pathways in GSVA between leading edge and other tumor region

Data S8. Enriched KEGG pathways of cancer epithelial cells from lymph node metastasis
